# Disentangling *Arachis* response to biotic and abiotic stress using multi-transcriptomics integration

**DOI:** 10.1186/s12870-026-08551-5

**Published:** 2026-03-13

**Authors:** Giulia Calia, Ana Paula Zotta-Mota, Manon Vidal, Sophia Marguerit, Ana Cristina Miranda Brasileiro, Patricia Messenberg - Guimaraes, Silvia Bottini

**Affiliations:** 1 Institut Sophia Agrobiotech, UMR 1355 INRAE/UniCA, INRAE, Université Côte d’Azur, 400 route des Chappes – BP 167, Sophia Antipolis Cedex, 06903 France; 2 Embrapa Genetic Resources and Biotechnology, Brasilia, DF 70770-91 Brazil

**Keywords:** Multi-condition omics integration, Plant responses to stress, Wild *Arachis*, Root-knot nematode, Drought stress

## Abstract

**Background:**

Peanuts are a fundamental legume in human diet, yet they are exposed to simultaneous and interacting biotic and abiotic stresses. Root-knot nematodes (RKN) and reduced water availability constitute a significant threat to this crop. Resistance signatures to each of those stresses have been identified in wild relatives such as *Arachis stenosperma* and *Arachis duranensis*. However, plant response to multiple stresses is very complex, requiring the activation of the appropriate signalling pathways to respond to all or by prioritising the response to one stress factor. Despite the experimental data availability of wild *Arachis spp.* subjected to RKN and/or drought stresses, the global regulome and crosstalk among biotic and abiotic molecular mechanisms have not yet been fully elucidated.

**Results:**

In this study, we applied HIVE, an integrative analytical framework, to study transcriptional responses in two wild *Arachis* species subjected to the root-knot nematode *Meloidogyne arenaria* and/or drought stress across six independent, unpaired experiments. We inferred a global regulome of biotic and abiotic response of two wild *Arachis* species from HIVE findings. The study of this gene regulatory network allowed the identification of novel regulatory mechanisms, specifically focusing on transcription factors and signaling pathways, potentially involved in *M. arenaria* and/or drought stresses response.

**Conclusions:**

Our results demonstrate that HIVE outperformed conventional meta-analysis approaches enabling the identification of novel and promising candidate genes potentially responsible for triggering effective defence responses to multiple stresses.

**Supplementary Information:**

The online version contains supplementary material available at 10.1186/s12870-026-08551-5.

## Background

From omics to multi-omics, we have witnessed a rapid growth in data generation accompanied by an increase in the multitude of experimental designs. These studies can provide a global understanding of the flow of information, by allowing the inference of complex interaction networks that link initial biological process to downstream functional consequences [1]. In the last decade, omics and multi-omics studies have been employed in several biological fields, including the study of plant immunity [2, 3]. Plants exhibit remarkable cellular plasticity during their growth and development, for instance, they are able to repair tissues after wounding or to regenerate entirely new organisms, as well as to reprogram cells to aid symbiotic interactions with other organisms or to counteract the attack of bio-aggressors [4]. Similar to many biological processes, the plant defence response mechanisms are composed of several physiological and molecular changes mediated by complex regulatory interactions. These processes occur at different biological layers (such as post-transcriptional, post-translational, translational and transcriptional changes), depending on the cells or tissues and the spatio-temporal scales. During infection, pathogen-derived proteins play a crucial role in reprogramming host biochemical processes, ultimately facilitating the infection's progression. Parasites modulate the immune response, cell signalling, MAPK and hormone signalling, vesicle trafficking systems, ubiquitination systems, metabolism or even plant growth [5]. As a counterpart, hosts use their own protein machinery to trigger defence mechanisms against the pathogen. In addition to biotic stresses, as sessile organisms, plants must endure abiotic stresses such as drought, high or low temperature, nutrient deficiency, salinity that can occur concomitantly to pathogens attacks. All these environmental conditions affect plant physiology causing several changes in molecular processes that can be non-adaptative or adaptative responses [6]. The former are direct responses to the stress, for instance protein structure changes caused by extreme temperatures or disruptions in enzyme kinetics and molecular interactions caused by toxic ions. The second category concerns responses that can improve stress resistance including the repair of stress-induced damage, the rebalancing of cellular homeostasis and the adjustment of growth [7, 8]. These stress-responsive mechanisms represent potential targets for crop improvement. Therefore, understanding how plants perceive stress signals and adapt to adverse environmental conditions both biotic and abiotic is critical for ensuring global food security.

Although in natural environments multiple stresses occur contemporarily, omics experiments mimicking these conditions are challenging to perform. Hence, most omics experiments examine responses to a single stress at a time and subsequently identify shared or stress-specific molecular signatures through comparative data analysis. The limitation of this approach consists in an underestimation of inter-conditions information yielding a very low number of molecular signatures modulated in response to multiple conditions. Consequently, there is an exponential trend in terms of volume and complexity of omics data that are currently under-exploited due to the lack of robust methodologies to successfully integrate data across heterogeneous experiments.

Wild peanut relatives (*Arachis spp*.) have evolved and adapted to a wide range of environments, resulting in extensive genetic diversity and high levels of resistance to several biotic and abiotic constraints. As a consequence, these species constitute a valuable reservoir of adaptive traits and resistance genes that can be exploited to improve stress tolerance and resilience in cultivated peanut and other crops. To identify those signatures, a previous study performed the comparative analysis of two wild *Arachis* RNA-Seq data upon root-knot nematode (RKN) *Meloidogyne arenaria* infection and/or drought stress, via meta-analysis [9]. However, this approach led the discovery of very few genes responsive to both stresses in both wild species. Furthermore, the complex network of regulatory interactions was not investigated at the time.

Lately, we developed HIVE (Horizontal Integration analysis using Variational AutoEncoders) to analyse at the same time unpaired multi-conditions transcriptomics experiments [10]. HIVE is particularly suitable when multiple conditions, each from a different experiment, need to be analysed jointly to capture common and/or specific molecular signatures. This peculiar scenario is very common in plant biology because multi-stress experiments are challenging to perform. Here we applied HIVE to the above mentioned dataset, comprising seven biological conditions and six distinct transcriptomic experiments, to perform multi-stress joint analysis in silico. We further demonstrated the advantages of performing the integrated analysis with HIVE compared with previously employed analytical methods. By inferring the global regulome of biotic and abiotic response of two wild *Arachis* species, we identified novel regulatory mechanisms potentially involved in this response and two novel resistance genes, which we validated *in planta*.

## Materials and methods

### Datasets collection

We collected RNA-sequencing data from six independent experiments, comprising roots of *Arachis duranensis* under biotic stress (*M. arenaria* infection) or abiotic stress (drought and desiccation stress), roots of *Arachis stenosperma* upon biotic (*M. arenaria* infection) and/or abiotic stress (*M. arenaria* infection and drought stress leads to a cross- stress), and their respective controls (not submitted to stress) (Table 1). For each genotype, *M. arenaria* infection was studied in a time-course experiment composed of three time-points, while drought and desiccation were available in a single time point. The cross stress (combined drought and *M. arenaria* infection) was available only for *A. stenosperma* and for a single time point. Bioinformatic analyses for gene expression quantification of each experiment has previously been performed and gene count matrices available at NCBI (Table 1). We collected three sample replicates for the drought stress “dry-down” and one sample replicate for the desiccation stress “dehydration” experiments for each *Arachis* species (Mota et al., 2021). For the biotic experiments, two sample replicates for each time point (three, six and nine days post- infection, dpi) for each plant species were collected, while for the cross-stress three sample replicates were collected only for *A. stenosperma*. With the respective controls for each experiment, we retrieved a total of 35 samples. Hereafter we will refer to the different stress conditions as “biotic stress” for *M. arenaria* infection, with “3/6/9 days post infection (dpi)" when considering time-points, as “abiotic/drought stress” only for the drought stress condition, while the desiccation condition will be expressively referred if considered. “Cross stress” will be used to refer at the combined condition of *M. arenaria* infection and drought stress in *A. stenosperma*.

**Table 1:** Summary of experiments used to construct the integrated dataset. For each condition, the plant species, the number of replicates and the original reference are reported

Plant Species	CTRL	3 dpi	6 dpi	9 dpi	Cross Stress	Drought (7 days)	Desiccation	References
*A.stenosperma*	2	2	2	2	-	-	-	[11]
*A.stenosperma*	3	-	-	-	3	3	-	[9]
*A.stenosperma*	1	-	-	-	-	-	1	[12]
total	6	2	2	2	3	3	1	
*A.duranensis*	2	2	2	2	-	-	-	[13]
*A.duranensis*	3	-	-	-	-	3	-	[9]
*A.duranensis*	1	-	-	-	-	-	1	[12]
total	6	2	2	2	-	3	1	16
global	12	4	4	4	3	6	2	35

### HIVE usage

For this study we applied HIVE to perform the integrative analysis of all the conditions [10]. HIVE suite is composed by three interdependent python scripts, each with a specific scope: data integration, latent space retrieval and quality assessments (yHIVE), correlation of latent features with phenotypic traits of interest (bHIVE) and gene selection and association to stress condition/s. We first used yHIVE script to obtain the latent space and intermediate results for evaluating the batch effect correction. Then, we applied bHIVE to further explore the reliability of latent space components and whether it was biologically informative. Finally, we applied gHIVE to extract the genes involved in single and multiple stress response of wild *Arachis* species.

### Dataset integration and preprocessing

We performed the integration step and the preprocessing following the yHIVE section of README file in the referring GitHub repository, “Plant-Net/HIVE”, specifying each argument accordingly and including the “-hz” and “—minmax” arguments in command line. The original structure and the learning epochs of the Variational AutoEncoder (VAE) from [10] were used. After using yHIVE to prefilter low-expressed and low-variance genes, the final integrated dataset contained 27,557 genes, whose expressions were min–max scaled, with the “—minmax” argument specification and then used to feed yHIVE to obtain the latent space composed of 80 latent features.

### Latent features’ reliability

To verify if latent features captured biologically meaningful information (e.g. association to studied phenotypic characteristics), we used yHIVE results for clustering metrics to assess the batch effect reduction in the latent space and then the bHIVE python script from HIVE suite. We compared clustering performances with raw data, PCA and t-SNE, applying a k-means clustering for *k* in a range from 2 to 10, as described in Supplementary Notes from [10]. We applied the default configuration for bHIVE (refer to the README file in the GitHub repository), to perform both point-biserial correlation and Kruskal–Wallis followed by the post-hoc Dunn test by specifying the phenotypic characteristics of interest. For the former we assigned, to not control samples, binary labels identifying the two pairs of plant species and type of stress (we included the cross-stress into abiotic stress group), using the”–pheno" argument. For the latter we considered all types of stresses and plant species separately only grouping time points in samples subjected to biotic stress.

### Gene extraction and association to condition

To extract genes majorly involved in the response to multiple stresses we used the gHIVE python script from HIVE suite. To also assign genes to a specific or multiple stress conditions, we first calculated the log2 fold-change of genes selected in the step before, using the DESeq2 package [14], then we used the gHIVE script in its default configuration (refer to the README file in the GitHub repository), specifying both “-ga” argument for association to condition and “-fc” to import the log2 fold-change values. We also used the “-M” argument with its “-cM” and “-fM” necessary arguments to define which association to merge, namely the time points in biotic conditions, and their final names.

### Comparative analysis

To compare HIVE findings with the meta-analysis we retrieved the list of genes with a common regulation (up or down-regulated) for both *Arachis* species from Mota et al. (2021) [9], and we run similar analysis for batch effect correction, gene selection, and functional analysis as in Calia et. al. (2025) [10].

### Functional analysis

The web-based Gene Ontology toolkit for Agricultural Community, AGRIGOv2 [15] was used to retrieve all the GO-terms associated with each gene in the *A. duranensis* genome. GO term enrichment was performed on the same website using the Hypergeometric statistical test and the GO terms list from Plant GO slim developed by The Arabidopsis Information Resource (TAIR) [16]. To retrieve information such as hormone-related genes and kinases, we performed protein annotation with MERCATOR4-v7 [17] web-based tool. First, CDS sequences were downloaded from PeanutBase database [18]. The obtained sequences accurately parsed to retrieve only the HIVE selected gene sequences are then used as input of MERCATOR4. Here we specified the type of sequence as DNA and we flagged the option to include Swissprot annotations. The list of NBS-LRR genes was retrieved from a previous publication [13], including the experimentally validated genes. The list of transcription factors was retrieved from PlantTFDB [19]. All the output files have been downloaded and then parsed with ad hoc python scripts.

### Orthologs analysis

To compare our list of genes, referring to *A. duranensis* PeanutBase [18] gene accession names, with previous studies in which a qRT-PCR on *A. stenosperma* genes was used to confirm their findings we had to set-up a tailored orthologs analysis. We first took both forward and reverse primer sequences (5’−3’) from two of the five publications used for screening [9, 11], and we performed a BLAST analysis with web-based blastn algorithm [20–22]. We choose the search set from NCBI Transcript Reference Sequences using the “*Arachis stenosperma* (taxid:217,475)” organism. Once obtained BLAST results, we applied following filters on alignments in order to retrieve our best hits: 100% of identity, 100% of query coverage and e-value < = 0.01. From the best hits we retrieved the corresponding protein sequences of *A. stenosperma* (genotype V10309) and downloaded the *A. duranensis* (V14167) proteome, both from PeanutBase. With these two sets of proteins, we applied OrthoFinder tool with default parameters [23] and finally retrieved *A. duranensis* genes belonging to the same orthogroups of the qRT-PCR validated *A. stenosperma* genes. With the correct gene accession name, we were finally able to screen the list of genes found by gHIVE for overlaps.

### Common and specific functional terms of HIVE selection

To functionally characterize the association between selected genes and conditions we performed two dedicated GO-term enrichment analysis as described in the paragraph before. We first retrieved the genes associated to all conditions, namely biotic and abiotic stresses in each *Arachis* species, and cross-stress in *A. stenosperma* and performed the first GO-enrichment analysis. Then we retrieved genes associated to one, and only one, condition of the above mentioned and singularly performed the GO-term enrichments. As a consequence, the enriched GO-terms can be shared between the different analysis, but the related genes will be surely different. In all analysis, we used the 0.05 p-value as threshold for enrichment.

### Cumulative distributions

We compared the gene selection of HIVE and meta-analysis, using the cumulative distribution of gene expression. From each method we extracted the genes associated to both specific conditions and to all the conditions as described in the previous paragraph and compared those distributions with the global expression in *A. duranensis*. The python numpy.histogram function was used to first bin the distribution and count the occurrences per bin, then we calculated the Probability Density Function (PDF) and consequently the Cumulative Distribution Function (CDF) with numpy.cumsum. To quantify the differences between the underlined distributions we used the Kolmogorov–Smirnov test (kstest module from scipy.stats python library).

### Hierarchical clustering of gene expression profiles

To visualize the expression profiles as heatmap and perform hierarchical clustering we used the “pheatmap” R package [24] using Spearman correlation as distance measure for genes clustering using as input the log2 fold-change calculated previously.

### Gene regulatory network (GRN) inference and analysis

We used GENIE3 R package [25] to infer the GRN, providing as “regulators” the list of transcription factors and as “target” the remaining genes. We retrieved the transcription factors for *A. duranensis* from Plant Transcription Factor Data Base (PlantTFDBv5.0) integrated in Plant Transcriptional Regulatory Map (PlantRegMap) [19, 26]. We used the Networkx python library to build-up and visualize the resulting GRN [27].

Specifically, we used the Networkx built-in function, “from_pandas_edgelist()”, to declare the regulatory and target genes from a pandas dataframe obtained by parsing GENIE3 output and the “DiGraph()” module to reconstruct a directed network. The visualization via Networkx is obtained by the built-in function “draw()” and specifying the node position passing to the function “spring_layout()” an indirect version of the previously obtained network.

We used the Leiden community detection method [28], implemented into the python library leidenalg that is built on the igraph library [29, 30], to find communities in our network and the library on the GRN with the “partition_type” parameter set as leidenalg ModularityVertexPartition. We then explored each community with ad hoc python scripts using methods for functional analysis illustrated in the previous paragraphs.

### Overexpression of NLRs in Arabidopsis

For “in planta” validation of NLR candidates, we used the 1,920 bp coding sequence of *AsTIR19* (Aradu.G29L) from *A. stenosperma,* previously cloned in the pPZP_BAR *Agrobacterium* binary vector, and successfully overexpressed in transgenic tobacco plants for *Sclerotinia sclerotiorum* resistance [31].

To identify the complete coding sequence of *AsHLR*, the Aradu.HLR71 gene model from *A. duranensis* was aligned with the four best BLASTn hits of *A. stenosperma* databases available on NCBI. The consensus sequence (1,296 bp) was synthesized and cloned (Epoch Life Science Inc., TX, United States), under the control of the *Arabidopsis thaliana* actin 2 promoter (ACT-2) and the nopaline synthase (NOS) terminator, at the *XhoI* restriction site of pPZP_BAR [32].

Both vectors were used for *A. thaliana* ecotype Columbia (Col-0) transformation using the floral dip method [33], and the overexpression of the transgenes in the OE lines at T2 generation was confirmed by qRT-PCR analysis [34].

### Assessment of the effect of NLR genes on RKN resistance

For the *M. incognita* bioassay, four- weeks old *A. thaliana* plants overexpressing *AsTIR19* (Aradu.G29L) and *AsHLR* (Aradu.HLR71) coding sequences were challenged with 500 juveniles (J2) of *M. incognita* as previously described [32]. Ten plants from each of the six Arabidopsis OE lines and the non-transgenic control (WT) were used, and after 60 days of infection (60 dpi), the number of eggs per gram of root (N° eggs/gr of root) was estimated in OE lines and WT plants, according to [35]. Statistical analyses were performed using one-way analysis of variance (ANOVA) to evaluate differences in nematode infection levels among the transgenic OE lines. The response variable analysed was the number of galls per gram of root tissue. When significant differences were detected, means were separated using Tukey’s test at a significance level of ntonino e*p*-value < 0.05. Data are presented as mean ± standard error (SE). All statistical analyses were performed using the python library StatsModels.

## Results and discussion

### Case study description

Wild species are known to be a good source of resistance genes, due to their high capacity to cope with different stresses. To identify novel resistance genes in different wild *Arachis* genotypes we studied: *A. stenosperma* highly resistant to the root-knot nematode (RKN) *M. arenaria* developing a hypersensitive response [36] and moderately tolerant to drought stress and *A. duranensis* showing the opposite behaviour [37]. We have selected 16 RNA-seq libraries for a total of 35 samples obtained in six different experiments (i.e. batches) investigating seven different biological conditions as from Mota et al. (2021) (Table 1). This integrated dataset was previously analysed by Mota et al. (2021) using meta-analysis. However, because of the complexity of the datasets, the meta-analysis yielded the identification of few genes responding to multiple stresses. Here, we analysed this dataset with HIVE [10], a deep-learning tool to perform multi-transcriptomics integration. HIVE is composed by three interdependent python scripts that we consequently used to obtain quality measures of integration (yHIVE and bHIVE) and a list of genes (yHIVE and gHIVE), responding to specific or multiple conditions. Using those genes identified by HIVE, we inferred a gene regulatory network with the model GENIE3 [25] which allowed the identification of two novel uncharacterized resistance genes.

### Latent features are associated with phenotypic characteristics.

**Fig. 1:**
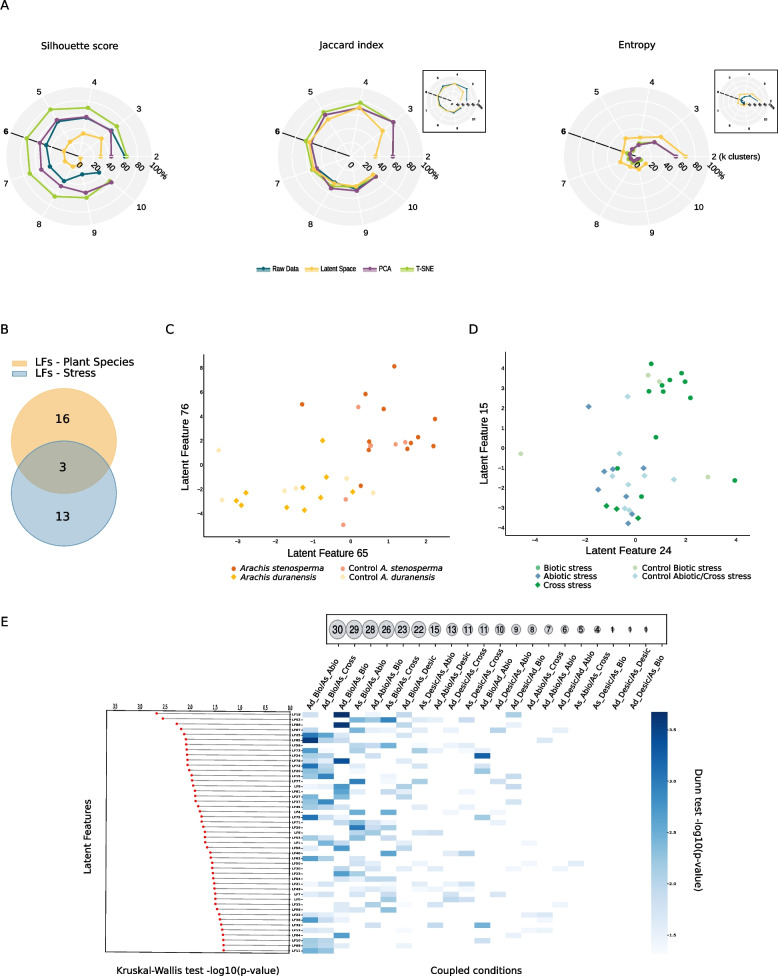
Latent space exploration. **A** Radar plot representing the Silhouette score, Jaccard index and Entropy of each method across different number of clusters (*k*) in K-means clustering. Insets are added to allow the comparison of metrics between HIVE latent space and raw data in cases where the plot trace for raw data is hidden from the trace of another method. **B** Number of latent features selected by the point-biserial correlation (*p*-value < 0.01) as the most correlated either with the type of stress (yellow) or plant species (light blue). Distribution of samples in the space of the top two correlated latent features with plant species (**C**) or with type of stress (**D**). The conditions are indicated in the corresponding legend. **E** Kruskal–Wallis test (on the left) and post-hoc Dunn test (on the right), application to pairs of conditions for each selected latent feature (“Desic” stands for desiccation stress and “Abio” refers only to the drought stress). On the top-right is represented the number of differing latent features according to the Dunn test results

First, we evaluated the consistency of using HIVE on this dataset. Therefore, we investigated the batch effect reduction in the latent space generated by the variational autoencoder (VAE), using the clustering metrics included in yHIVE. We obtained results for 2 to 10 clusters and compared the samples belonging to each cluster with the original batches using some of the obtained metrics like Silhouette score, Jaccard index and entropy. We applied the same clustering strategies on the raw data and upon dimensionality reduction using standard techniques like PCA and t-SNE. Strikingly, the Silhouette score and the Jaccard index on the HIVE latent space show the lowest values while entropy show the highest, independently on the number of clusters in the chosen range, compared to the correspondent scores obtained on raw data, PCA or t-SNE, confirming previous findings [10], (Fig. 1A and Supplementary Table 1).

Once we established the correction of batch effect in the latent space, we explored the latent features to study whether they capture patterns associated with the different phenotypic characteristics in the dataset. First, we studied the two main characteristics, namely the type of stress (biotic or abiotic) and the type of plant species (*A. duranensis* or *A. stenosperma*). By using bHIVE point-biserial correlation, we calculated the most significant latent features correlating either with the type of stress or the species. Among the 80 latent features, we found 19 features correlated with plant species and 16 with biotic/abiotic stress (Fig. 1B). Interestingly, three of them were found significantly correlated to both characteristics. Then we plotted the two most significative latent features associated with each phenotypic characteristic and we observed samples grouping accordingly (Fig. 1C and D). Samples from the experiment with the cross stress (*M. arenaria* infection and drought at the same time) cluster together with the other samples of abiotic stress. The bHIVE Kruskal–Wallis followed by the post-hoc Dunn test, was then used to compare pair-wisely the conditions and counting how many latent features are associated to the discrimination of each pair of experimental condition (Fig. 1E). *A. duranensis* biotic and *A. stenosperma* drought showed the highest number of latent features (30 latent features) associated to their difference followed by *A. duranensis* biotic and *A. stenosperma* cross and *A. duranensis* biotic and *A. stenosperma* biotic (29 latent features and 28 latent features respectively) as expected therefore confirming the validity of the latent features to resume the multiple phenotypes of the datasets. Interestingly, the similarity of *A. stenosperma* drought and *A. stenosperma* cross was also confirmed by this analysis because only one latent component was found associated with differentiating them. Due to the presence of only one replicate for desiccation stress and the very few features discriminating this experimental condition from the others, we decided to not consider those samples for further analysis.

Overall, these results show not only that the latent features are free from batch effect but also that they capture the phenotypic characteristics of the dataset even when poorly represented.

### Comparative analysis with previous findings and state of the art tool

**Fig. 2:**
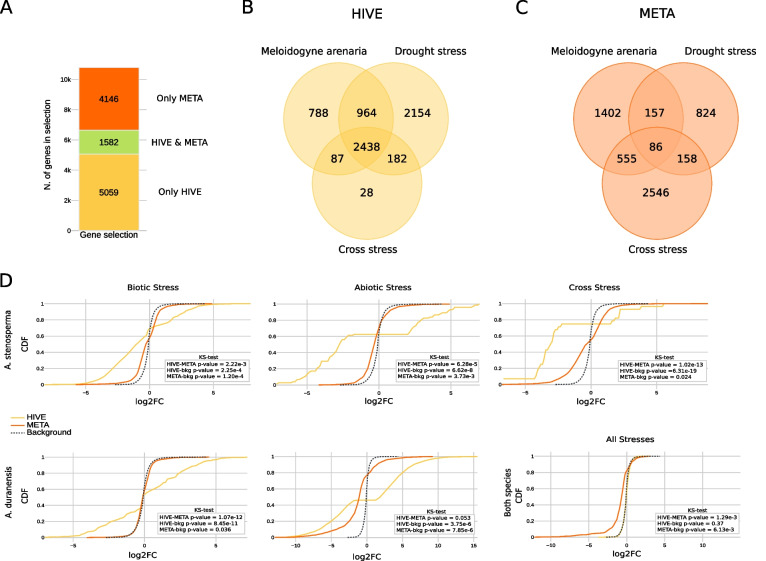
Comparison of HIVE and meta-analysis. The term “META” in the figure refers to meta-analysis results. **A** Bar chart representing HIVE gene selection, META gene selection and their agreement. **B**, **C** Venn diagram representing the agreement of three sets of genes from the HIVE and META selections, respectively. Those associated with *Meloidogyne* arenaria infection (for both peanut species), those associated with drought stress (both peanut species), and those associated with the cross stress (only *Arachis stenosperma*). **D** Displays the cumulative distribution of genes in the two peanut species, associated with specific or all conditions for both HIVE-associated genes and meta-analysis ones. On the bottom right of each triplet of cumulative distributions is reported the *p*-value (significance threshold < = 0.05) for the Kolmogorov–Smirnov test for each paired comparison

To study whether the use of HIVE is suitable for performing the integrative analysis of the dataset under investigation, we compared the results of gHIVE at the stage of gene selection, with the meta-analysis conducted in the study of Mota et al. [9]. First, we compared the number of genes found by the two methods. HIVE and the meta-analysis found a similar number of genes 6,641 and 5,728 respectively (Supplementary Table 2, Fig. 2A). By inspecting the overlap among these lists of genes selected by each method, we observe that overall agreement is very low with just 1,582 genes (4,2% of HIVE selection). With a similar aim, we performed GO term enrichment analysis to study the biological mechanisms in which selected genes by each method are involved (Supplementary Table 3). The two tools showed similar number of enriched pathways (26 for HIVE and 29 for the meta-analysis), but few in common (only nine). However, common enriched GO terms are involving important pathways for plant defence response like “kinase activity”, “response to stress”, “response to stimulus” and “response to biotic stimulus”. Furthermore, exploring the enriched GO terms found only by HIVE we can find “photosynthesis”, “nitrogen compound metabolic process” as biological processes and “thylakoid” as cellular component. Photosynthesis, as essential molecular process has in fact a central role in plant defence response to biotic stress like secondary metabolites production and stress-growth prioritization [38, 39], and in abiotic stress involving signalling pathways and chemical compounds modulations [40]. Part of the photosynthetic system are the thylakoids, often targets of phytopathogen effector proteins and sites of Reactive Oxygen Species generation in chloroplasts [38, 41]. Finally, nitrogen is one of the essential nutrients in plant growth and crop yield. Its uptake can determine the establishment of a bio-aggression or its defeat and nitrogen ions coordination lead to pH and homeostasis regulation under abiotic stress response [42, 43]. Altogether, these enriched GO terms confirm an overall reliability of HIVE selected genes.

An important aspect in integrative analysis is the number of signatures common to all conditions and specific to one. Using gHIVE to also associate selected genes to stress conditions, we found over 28% (1,919 genes) of the genes responding to all conditions (Supplementary Table 4). The meta-analysis of the biotic, abiotic and cross-stress separately performed in the original study included 1,224, 2,200 and 3,345 genes, respectively [9]. Importantly, the overlap of these three lists is accounting of only 1.5% of the total common genes (86 genes) (Fig. 2B, C). The small overlap of the three lists obtained from each separated study represents the major limitation of the meta-analysis since the number of common genes usually decrease with the increase of the number of studies considered.

Moreover, due to the constraints imposed by the meta-analysis, most of the genes found are either associated to biotic or abiotic or cross-stress condition separately, thus suggesting that genes involved in response to more than one stress are not identified by the method. HIVE found a more balanced number of genes associated specifically either to biotic or abiotic conditions, while very few associated specifically to cross-stress. This can be expected given the high number of shared signatures between cross-stress and the two single stresses, and allow us to fairly reconstruct the crosstalk of *Arachis* defence response by the in silico integration of single stress experiments (Fig. 2B, C and Supplementary Fig. 1).

Finally, we investigated the level of regulation of genes found by each method as specifically selected in response to one condition or shared to all stresses. From the cumulative distributions showed in Fig. 2D, we can observe that the genes selected by HIVE as specific of each condition, show the most extreme values of modulation when compared to the background, either up or down regulation, with the most significative p-value compared to the previously selected genes and background (Supplementary Table 5). This result highlight that the genes found by HIVE as responsive specifically to one condition have an overall level of regulation which is stronger than the genes selected by meta-analysis for the same category. Concerning the shared signatures, namely genes modulated in all conditions, we expect a milder level of regulation compared to specific signatures. Overall, independently by the selection method, we observe that the cumulative distributions of those shared signatures are closely distributed to the background. Since in HIVE model there are no constrains on expression values, the model is able to also capture shared mildly expressed signatures missed by the meta-analysis. Those results corroborate the suitability of HIVE to analyse this dataset.

### Specific and common molecular *Arachis* response to biotic and abiotic stresses

**Fig. 3:**
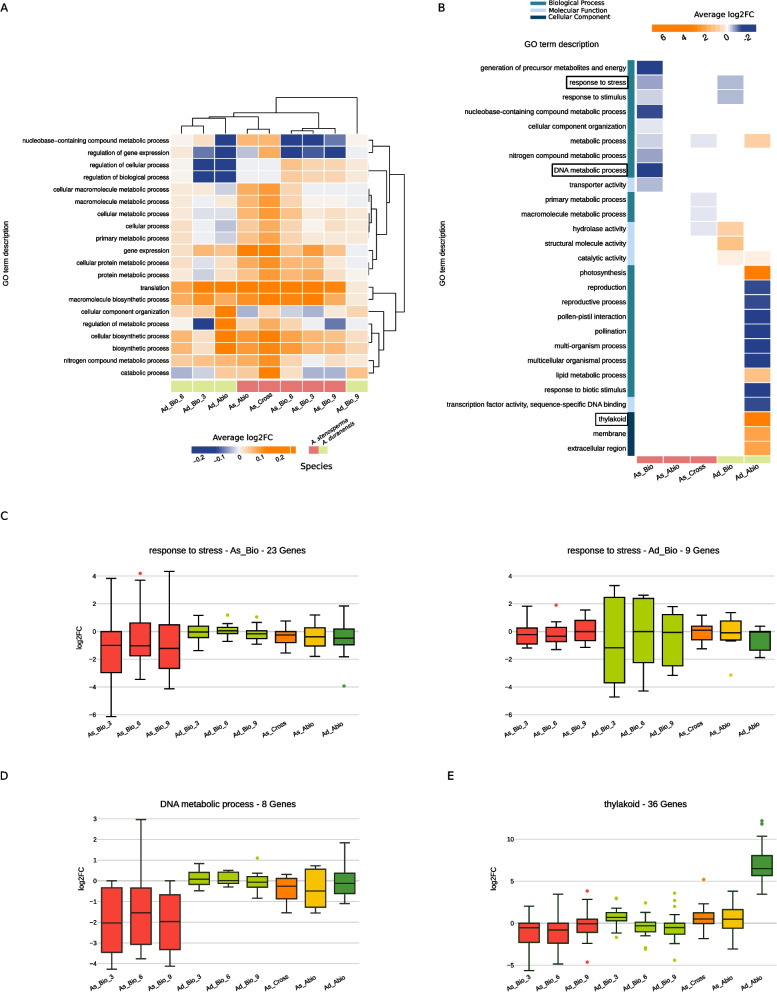
Functional analysis of genes associated with common or specific conditions. **A** Log2 fold-change of genes, in HIVE selection, associated with all conditions. **B** Enriched GO terms found in genes from HIVE selection associated with specific conditions. Color-scale represents the average log2 fold-change of genes associated with the respective term. Black squares correspond to the terms plotted in the following: Zoom-in into the log2 fold-change of genes related to C response to stress in biotic conditions from the two peanut species; **D** DNA metabolic process; **E** thylakoid. When the biotic conditions are considered as separated time points, the “_3”, “_6” and “_9” notation refers to 3, 6, and 9 days post infection

In the previous paragraphs we showed that HIVE identified the highest number of common genes responding to all condition. Therefore, we decided to inspect the functional pathways associated to common and specific signatures to understand which biological processes are commonly modulated in response to both stresses and in both species and which are very specific of only one condition. To analyse the common signatures, we selected the 1,919 genes modulated in all conditions and performed GO term enrichment analysis and reported for each condition the average expression values of genes belonging in significantly enriched terms (Fig. 3A and Supplementary Fig. 2). Interestingly, by clustering the conditions based on those average expression profiles, we observe that the clusters correspond to the similarity of the phenotypes. Strikingly, very few enriched terms associated to the selected common genes are modulated similarly in all condition, but most are modulated depending on the stress and/or genotype. This result suggests that despite the same genes and terms are involved in response to both biotic and abiotic stress in both species, their activation or repression can be specific of each condition. For instance, while genes involved in translation and macromolecule biosynthetic process show the tendency to be upregulated in all conditions, regulation of gene expression and nucleobase-containing compound metabolic process show overall down-regulation in abiotic stress and in biotic stress for *A. duranensis* at three dpi together with all time points of biotic infection for *A. stenosperma* and upregulation in the other conditions while regulation of metabolic process is down-regulated only in three dpi for *A. duranensis* and slightly in nine dpi for *A. stenosperma*. Similarly, when we inspected the expression trends of transcription factors (TFs), belonging to important families for biotic/abiotic stress response like WRKY, MYB, SBP, bHLH, bZIP, NAC, and ERF [44–46] (Supplementary Fig. 3 A), we observe a stress/genotype dependent modulation of their expression as well as for genes involved in phytohormones signalling pathways (Supplementary Fig. 3B). For example the gene Aradu.KVT1I (ethylene related gene), is negatively regulated in A. stenosperma infected by M. arenaria but is positively regulated especially in the cross stress condition, following pivotal findings in Mota et al., 2021 [9]. Afterwards, we inspected the specific signatures by performing the same analysis on the genes that were found to be modulated specifically in only one condition (Fig. 3B). Strikingly, although the genes selected for each condition are different, we can observe that the same terms are found enriched in multiple conditions, suggesting that the same pathway is modulated in response to both stresses and species but different genes are involved. For example, the term “response to stress” is enriched in response to biotic stress in both genotypes, despite 23 genes are modulated in *A. stenosperma* and 9 in *A. duranensis* (Fig. 3C). Other terms are specific of one unique condition, such as DNA metabolic processes, whose correspondent 8 genes are downregulated in *A. stenosperma* during biotic stresss (Fig. 3D); or thylakoid cell compartment which shows up regulation of 36 genes only in drought stress for *A. duranensis* (Fig. 3E). By focusing more in detail on genes modulated only in biotic stress (788 genes) and only in abiotic stress (2154 genes), we could explore the differences between the three set of genes in terms of TF families and phytohormones pathways involved. We found TFs from WRKY, ERF, bHLH and MYB families across set of genes modulated in all conditions and those modulated in one of the two types of stresses. On the other hand, NAC and bZIP families are present only in response to abiotic stress or to all conditions (Supplementary Fig. 3 C). Those two families of TFs are shown to be mainly involved in response to abiotic stresses both in peanuts and in other plants [47–50]. The differences in proportion of genes associated to these families in the three sets, suggest that different genes are modulated in similar molecular processes depending on the stress condition/s. Similarly, for phytohormones related genes, we observe the presence of jasmonic acid, abscisic acid and brassinosteroids just for the set of only abiotic stress related genes or in all conditions, while the other phytohormones are present in different proportions across the three sets of genes. Importantly, abscisic acid and brassinosteroids, are typically modulated in abiotic stress response [9, 51] (Supplementary Fig. 3D). Altogether these results show that core pathways can be regulated differently in response to the different conditions, and that specific responsive genes can be involved in the same pathway triggered in response to multiple conditions.

### The integrative analysis identified novel signatures in the main hormone signalling pathways, kinases, and resistance in response to biotic and/or abiotic stress

To further elucidate the functionality of HIVE genes involved in plant responses to multiple stresses, we inspected the 6,641 genes found by our integromics approach searching for genes involved in the main phytohormones signalling pathways, kinases and resistance genes.

**Fig. 4:**
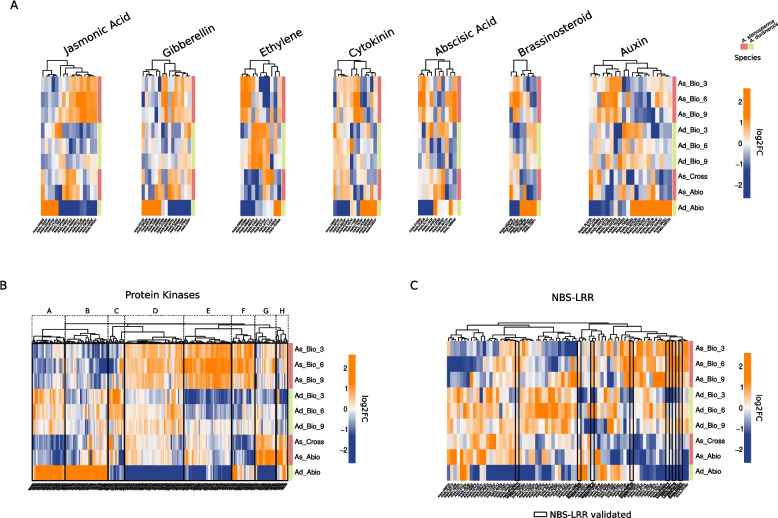
HIVE identified genes involved in the main hormone signalling pathways, kinases and resistance genes in response to stress. **A** Heatmaps of gene expression in each set of annotated phytohormones. **B** Heatmap of gene expression for annotated protein kinases found in HIVE selection, black squares represent clusters of gene expression (**A**-**H**). **C** Heatmap of gene expression for NBS-LRR genes found in HIVE selection; the black squares represent the experimentally validated NBS-LRR by Mota A., et al., 2018. The “_3”, “_6” and “_9” notation refers to 3, 6, and 9 days post-infection

Ninety-three genes were identified belonging to seven hormone signalling pathways, three directly connected with abiotic/biotic stress response (abscisic acid, jasmonate and ethylene) and four growth-promoting hormones (gibberellins, auxins, cytokinins and brassinosteroid). Considering the pivotal importance of plant hormones in mediating the regulation of stress responses, we focused on studying the expression profiles of these genes using a hierarchical clustering approach. Overall, we observe that these genes are activated or inhibited with very different patterns depending on the stress and the plant species (Fig. 4A). As expected, genes involved in the jasmonic pathway are mainly activated in *A. stenosperma* in response to nematode infection, corroborating the widely-known role of this signalling pathway in biotic stress response for plant protection [52–54]. Brassinosteroids are activated/inhibited according to the stress applied and plant species specific manner. Genes related to growth hormones, gibberellins and cytokines, show similar expression profiles, while some are activated in both stresses and plant species, and others show a more specific pattern. Interestingly, genes in the ethylene pathway are strongly activated in *A. duranensis* and more mildly in *A. stenosperma* during RKN infection. Finally, genes in the abscisic acid and auxin pathways show a very heterogeneous expression pattern, with an overall activation in response to drought mainly for abscisic acid-related genes. Previous analysis of the separated conditions showed that the jasmonic acid pathway was prevalently induced during biotic interaction, while abscisic acid upon abiotic stress [9]. The integrated analysis allowed to identify other important hormone pathways involved in defence response and their different modulation depending on the plant species. Notably, by literature screening of works related to the type of stresses/genotypes investigated in study we found 13/93 phytohormone related genes validated by qRT-PCR from Vinson et al., 2017, Guimaraes et al., 2015 and Mota et al., 2021 [9, 11, 12]. Results of the analyses performed to retrieve a comparable list of genes from qRT-PCR primers are reported in Supplementary Table 6 following data FAIRness principles [55] (refer to Materials and Methods section for further details).

Among HIVE-selected genes, we have found 219 genes coding for kinases, which are sensors for a variety of signals like phytohormones and small peptides, facilitating intercellular communication [56–58]. By inspecting their transcriptional profiles, we identified 8 clusters (Fig. 4B). Three clusters (A, E, G) group kinases induced in a stress and plant species-specific fashion (biotic—*A. duranensis*, biotic—*A. stenosperma*, abiotic—*A. stenosperma*, respectively). Kinases in cluster D are activated in response to biotic stress in both plant species. The remaining four clusters bear genes showing modulated activation in multiple stresses and plant species at the same time. Also in this case, we found 1 kinase related gene validated by qRT-PCR in Vinson et al., 2017 and 28 genes associated *to Arabidopsis thaliana* kinases orthologous groups by Martins et al., 2022 [59] (Supplementary Table 6). Moreover, among the broader list of genes retrieved by gHIVE we found correspondence of other 108 genes from the previous five publications and Mota et al., 2018 [13], both investigating peanut defence response to either biotic, abiotic or cross stress conditions. Notably, we could have confirmed the presence in our list of an endochitinase gene, Aradu.4196P, previously validated in planta from Mota et al., 2021 [9], and shown to be important in *A. thaliana* for tolerance of both nematode infection and drought stress.

Other key players of plant immunity are resistance genes (R-genes) [56]. They code for the R-proteins, which have a main role in recognizing secreted pathogen-specific proteins (effectors) and activate a powerful, pathogen-specific immune response, which often includes transcriptional reprogramming [60]. Most R genes in plants encode nucleotide-binding site leucine-rich repeat (NBS-LRR) proteins [61]. This large family is encoded by hundreds of diverse genes per genome and can be subdivided into the functionally distinct TIR-domain-containing (TNL) and CC-domain-containing (CNL) subfamilies [62]. Despite being very difficult to find due to their very low expression [63], HIVE identified 79 NBS-LRR genes whose expression profiles are reported in Fig. 4C. Conversely, the meta-analysis only found 55 NBS-LRR, reinforcing the utility to perform integrated analysis using HIVE. Overall, we observed for the NBS-LRR genes similar expression patterns as for the kinases: either they were induced upon specific stress-plant species, or showed heterogeneous patterns, showing modulations in multiple conditions that were not previously found by non-integrated analysis methods [13]. Of note, seven of them were previously validated by qRT-PCR assays [13], reinforcing the reliability of the results found by HIVE.

In conclusion, our analyses identified novel genes triggered in each different condition, linked to main hormone signalling pathways, kinases and R-genes in response to different stresses in comparison to meta-analysis.

### The global gene regulatory network allowed to study the regulatory processes induced in wild *Arachis* by biotic and/or abiotic stress

**Fig. 5:**
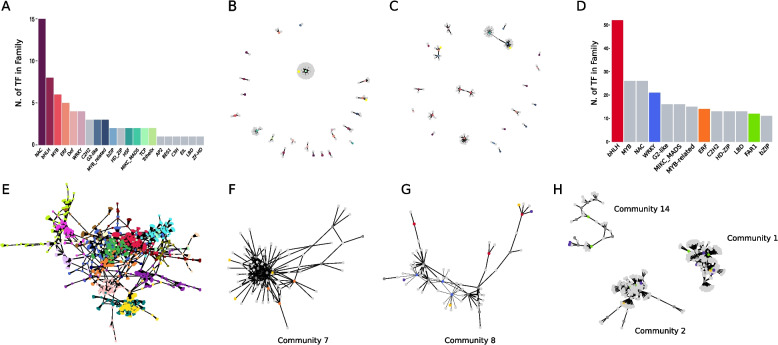
The integrated analysis allowed the inference of a complex gene regulatory network induced by biotic and/or abiotic stress. **A** Number of transcription factors from HIVE selection with predicted binding motif in PlantRegMap. **B** GRN inferred by GENIE3 imposing as “regulators”, the TF found by HIVE and PlantRegMap, and as “targets”, genes that are not TFs (after having removed all pairs not in PlantRegMap). Nodes are coloured according to A. **C** Same as B but with gene selection from the meta-analysis. **D** Number of transcription factors in families with at least 10 representatives in HIVE selection. **E** The largest connected network from the GRN inferred by GENIE3, imposing as “regulators”, the TF found by HIVE and not in PlantRegMap, and as “targets”, genes that are not TFs. Colours correspond to the 19 communities found by the Leiden algorithm. Zoom in on community n. 7, orange nodes correspond to transcription factors of the family ERF. Zoom in on community n. 8, bright blue nodes correspond to transcription factors of family WRKY, and red nodes to transcription factors of family bHLH. H) Zoom in on communities n. 14, 1, and 2. Bright green nodes correspond to transcription factors of the family FAR1. Dark grey nodes represent all the other transcription factors in the network not belonging to the family/families of interest, dark yellow nodes correspond to genes associated with phytohormones, violet nodes indicate NBS-LRR genes, and all the light grey nodes are other categories of regulated genes

Plant responses to stress are controlled by a complex gene regulatory network (GNR). Therefore, we focused to study the transcription factors (TF) – targets relationship. Using PlantRegMap [26] we found 69 TFs among the HIVE selected genes distributed in 21 families (Fig. 5A). We inferred the GNRs by considering those 69 TFs as the “regulators” and the remaining 6,287 genes that are not TFs as targets. By using the GENIE3 model [25] and selecting edges with a weight > = 0.09 (see methods), we inferred 2,214 connections. Then we filtered out the pairs not in agreement with the prediction from PlantRegMap, obtaining 22 TFs and 127 interactions (Fig. 5B). Interestingly, by inspecting those pairs we have found two TFs regulating genes involved in phytohormone pathways. The bZIP TF Aradu.C0RFP and the ethylene-related gene Aradu.MA5U7, with uncorrelated expression profiles (Supplementary Fig. 4 A) and the ERF- TF Aradu.K41I0 with the gene Aradu.1C9UI involved in the jasmonic acid pathway, showing correlated expression profiles (Supplementary Fig. 4B). We used the same procedure to infer the network on the genes selected by the meta-analysis. Among the 379 TFs in the meta-analysis, 76 are also present in PlantRegMap, yielding 5,349 genes as putative targets. Applying the same threshold, we selected 2,919 pairs. By filtering out the pairs not in agreement with PlantRegMap we obtained 23 TFs and 86 interactions (Fig. 5C), 32% less than the pairs found with HIVE genes. These results suggest that more real direct targets can be inferred from HIVE genes.

To reconstruct a more general regulome, we first extracted all the TFs among the genes selected by HIVE analysis (Supplementary Table 7). Among the families with more than 10 TFs found by our analysis we retrieved families with known pivotal roles in development, plant growth and stress responses (Fig. 5D) [25, 64]. Then we inferred another network using the same strategy, from the 354 TFs in HIVE selection, we removed the 69 used in the previous network for a total of 285 TFs as “regulators” and the 6,287 genes as “targets”. By selecting the top 2,500 pairs, we obtained a big connected community and several isolated interactions including 224 TFs and 1,410 targets (Supplementary Fig. 5). We focused on the main network for further investigations, and we applied the Leiden algorithm to find communities of genes densely connected [28]. Leiden community detection is a fast and accurate method that allows us to find well-connected and refined partitions of the network. This algorithm considers the modularity to identifies groups of nodes (communities) in a graph that are more densely connected internally than with the rest of the graph [65]. Therefore, in the present case, since the network is a GRN, a community represents a set of TFs which share more targets within each other than with the TFs in the rest of the GRN and using the “guilt-by-association” principle, they may share functions or pathway involvement. We obtained 19 communities as shown in Fig. 5E. Among them we explored 5 communities showing a higher number of phytohormones related genes, resistance genes and regulators belonging to important transcription factor families for plant stress response. First, we quantified the number of TFs belonging to the different families in each community (Supplementary Table 8), then we started to inspect community 7, which shows the highest percentage of phytohormones related genes with respect to the total number of genes in the community (Supplementary Table 9). These genes are involved prevalently in the jasmonic and abscisic acid pathways (Fig. 5F). Interestingly, this community also presents a high percentage of TFs belonging to the Ethylene Responsive Factors (ERF) family. The *crosstalk* among these three hormone pathways was already suggested in the previous publication related to these data and suggests a role of community 7 genes in tolerance mechanisms of wild *Arachis* for cross stress response [9]. The expression patterns of these genes and TFs are very similar among each other, all showing high activation in *A. stenosperma* under RKN infection, but different patterns in the other conditions. Aradu.LD7BF and Aradu.3P75R (two TFs), are highly repressed in *A. duranensis* in the earliest time point of *M. arenaria* infection. Aradu.E2TII and Aradu.XW8AZ (TF and jasmonic acid-related gene) are strongly repressed during drought stress in *A. duranensis*. Interestingly, Aradu.EQ5J6 (abscisic acid-related gene) is downregulated in both, early time-points of nematode infection and in drought stress condition in *A. duranensis.* Finally, Aradu.WB9GS (abscisic acid-related gene) shows a different modulation with a mild activation during drought stress in *A. duranensis* (Supplementary Fig. 6 A). Despite their enrichment, we did not observe a direct interaction between the ERF and hormone-related genes involved in jasmonic and abscisic acid pathways. This suggests that other intermediary TFs and genes intervene to regulate this complex *crosstalk,* providing novel potential key interactions to further inspect.

The second most enriched communities in genes related to phytohormones pathways are communities 8 and 2. In community 8 we found TFs belonging to stress related WRKY and bHLH families and also stress-responsive related genes. This suggests a role for genes in community 8 related to developmental processes affected by the stress response [66, 67], therefore, we screened this community for NBS-LRR genes (Supplementary Table 10) and found two members as direct targets of TFs in the two enriched families: Aradu.180Q6 (WRKY- TF) – Aradu.NP598 (NBS-LRR) and Aradu.X5F2F (bHLH-TF) – Aradu.HLR71 (NBS-LRR) (Fig. 5G). By inspecting their expression profiles, Aradu.NP598 showed a different pattern compared to the other selected genes in the community, with a strong repression in *A. stenosperma* after 6 and 9 days of *M. arenaria* infection, while Aradu.HLR71 showed mild activation in *A. stenosperma* independently of the stress, with similar expression profile to the putative TF regulator, Aradu.X5F2F (Supplementary Fig. 6B). In community 2 the most represented family of TFs is the far-red-impaired response 1 (FAR1), which is present with the highest percentage also in community 1 and 14. Intriguingly, community 1 bears the highest percentage of NBS-LRR genes. Therefore, we decided to analyse those three communities altogether (Fig. 5H). This family of TFs was shown to be implicated into pod development and abiotic stress response in *A. hypogaea* [68, 69]. Furthermore, we have observed high percentage of gibberellin related genes, whose modulation was shown to be important for controlling pod size [70], highlighting a common role of genes in community 1, 2, and 14. In the three communities we observe NBS-LRR genes either as direct targets or as secondary targets of FAR1- TFs, suggesting a possible regulatory role for this family in R-gene mediated *Arachis* response to stress like already evidenced in *Arabidopsis thaliana* [71]. We also observed that, in community two NBS-LRR genes, Aradu.RT9LH and Aradu.G29LA, are targeted by the same WRKY- TF (Aradu.VL3IZ), which is a target of a FAR1-TF (Aradu.1I5W4). The expression profiles of the FAR1 -TF and the two NBS-LRR is opposite in all conditions, with Aradu.G29LA showing higher differences compared with Aradu.RT9LH, suggesting an opposite activity mediated by the intermediary WRKY- TF (Supplementary Fig. 6 C). FAR1-TFs and WRKY-TFs are already shown to be interacting for modulation of plant defence response either to biotic or abiotic [72–74] stresses both in peanut and other plants, furtherly confirming the reliability of these 3 communities of genes in our inferred GRN.

Overall, the inferred GRN highlighted signatures with pivotal roles in stress responses and improved our understanding of the *crosstalk* among the different strategies used by plants to respond to diverse stresses.

### Plant functional validation of novel NBS-LRR genes

Among the NBS-LRR genes found in the previous analysis, we selected two genes to be validated in planta: AsTIR19 (Aradu.G29LA- G29), in community 14, and AsHLR (Aradu.HLR71), in community 8.

For in planta validation of these NLR genes in response to nematode infection, we produced six A. thaliana overexpressing lines (OE) for each gene, which were challenged by the RKN *M. incognita*.

**Fig. 6:**
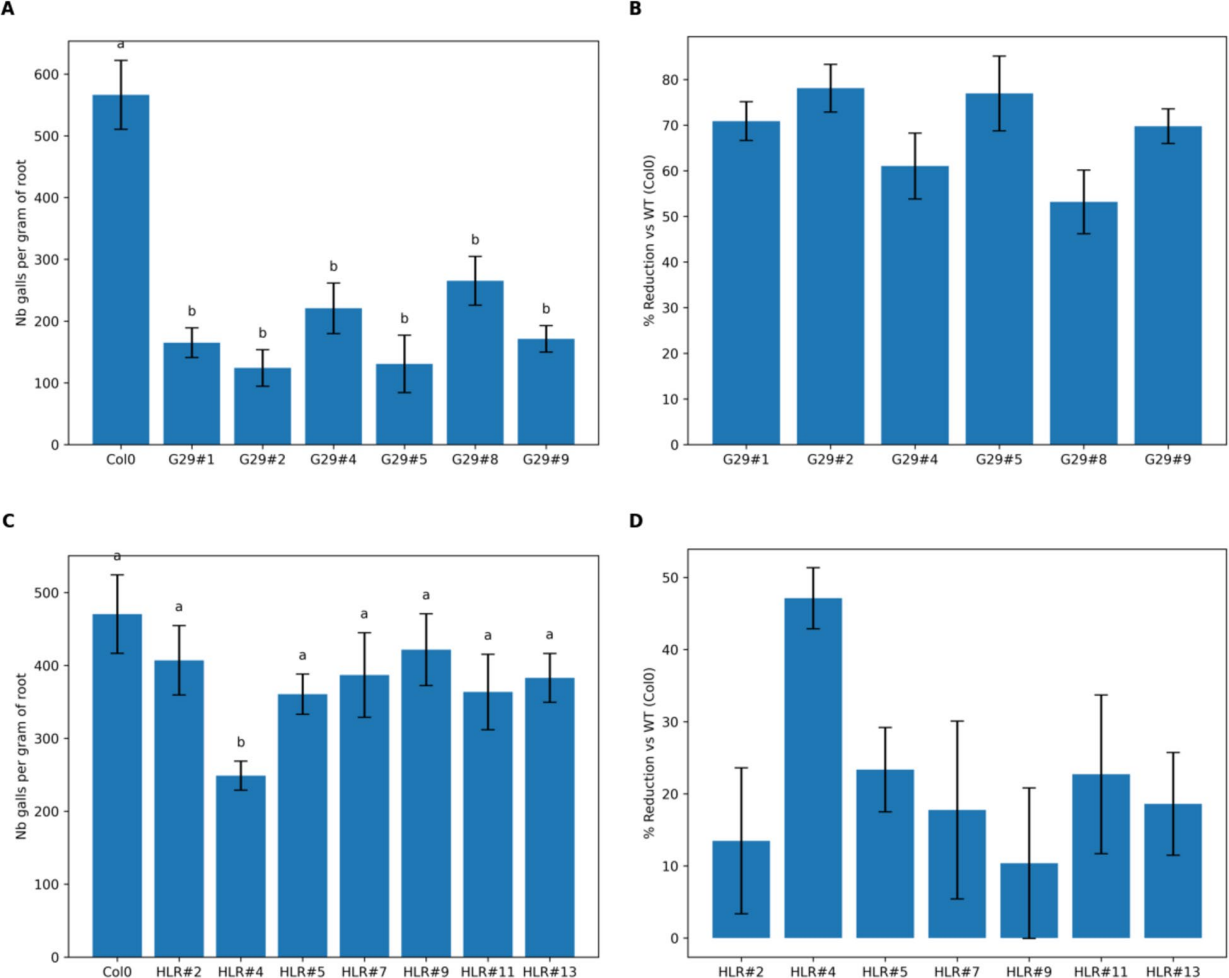
Functional validation of candidate transgenic events against Meloidogyne incognita. **A** Infection levels expressed as number of galls per gram of root in AsTIR91-OE lines- G29 #1, 2,4, 5, 8, 9 and WT; **B** percentage reduction of infection in AsTIR91-OE lines relative to WT plants; **C** infection levels expressed as number of galls per gram of root in AsHLR -OE lines: HLR 2, 4, 5, 7, 9, 11,13 and WT; **D** percentage reduction of infection in HLR lines relative to WT plants. Bars represent mean ± SE. Different letters indicate significant differences according to one-way ANOVA followed by Tukey’s HSD test (*p*-value < 0.05)

For AsHLR, one-way ANOVA revealed significant differences among genotypes (*p*-value = 0.0338). Post-hoc Tukey analysis indicated that line HLR#4 showed a significant reduction in infection compared to WT, while the remaining lines were not statistically different from WT (Fig. 6A). The overexpression of AsTIR19 in Arabidopsis showed a significant reduction in nematode infection compared to WT plants (one-way ANOVA followed by Tukey’s HSD test, p-value < 0.05), with infection reductions ranging from 53 to 78% relative to WT (Fig. 6B). These results suggest that, both genes, alone or in association with other defence genes, have the potential to increase M. incognita resistance in transgenic plants.

Our strategy allowed to identify two previously uncharacterized resistance genes against M. incognita from wild Arachis and functionally validate them in planta.

## Conclusions

Peanut is one of the most economically significant legume crops worldwide, serving as a key source of vegetable oil and protein in human diet. Reduced water availability, and pathogens spreading are great threatens to peanut productivity. These climate-induced stresses rarely occur in isolation, making their combined biological effects difficult to understand using conventional single-stress studies. The interaction between abiotic and biotic stresses in plants presents significant socio-economic and scientific issues, particularly in the context of climate change. Ensuring sustainable future food production requires a comprehensive understanding of the crosstalk and trade-offs resulting from combined abiotic and biotic stress impacts on plant growth and defense. In this study, we have shown how multi-transcriptomics integration with HIVE can elucidate the complex mechanisms involved in multi-stress response from single-stress experiments. HIVE is an unsupervised model that uses a variational autoencoder to remove the batch effects by creating a compressed representation of the original data called latent space. We have showed that this novel representation captures the salient patterns describing the experimental phenotype.

We also demonstrated that HIVE selects more genes implicated in pivotal molecular processes involved in plant response to stress compared to the meta-analysis, originally employed in the study of Mota et al. [9]. By studying the patterns of expression of these genes we showed how these pathways are modulated depending on the different phenotypes. Indeed, HIVE selected genes that are exclusively activated or repressed upon one stress in a particular plant species, but also more complex patterns such as genes modulated differently in the studied conditions. These results can help to better understand plant response to different stresses and constitute a useful resource of potential candidate genes for engineering broader stress tolerance in peanut. We also showed that by applying the network inference model GENIE3 [25] on genes selected by HIVE, we were able to reconstruct a comprehensive interactome of *Arachis* in response to *M. arenaria* infection and drought stress. We also showed that the candidates provided by HIVE enabled to reconstruct a network with more reliable connections than by using genes selected by the meta-analysis. The study of the inferred network allowed to better understand the crosstalk in response to biotic and abiotic stress conditions and to identify two novel NBS-LRRs with a potential role in resistance against RKN infection. Since those two genes were not identified by the previous work with the meta-analysis, those findings reinforce the powerful of our integrative approach coupled with GRN inference and improve the panel of resistance genes known for *Arachis*.

Overall, we provided novel analysis tools to study multi-conditions transcriptomics data for plant-stress response which can be applied to other phytopathosystems. We also provided improvements in our understanding of the mechanisms set up by *A. stenosperma* and *A. duranensis* upon biotic and abiotic interactions. Further investigations of our findings will allow the identification of important signatures that will enable the engineering of durable disease resistance in plants.

## Supplementary Information

Supplementary Material 1. Supplementary Table 1 K-means clustering performances. The clustering performances for the four dataset setups (Raw Data, Latent Space, PCA, t-SNE). Calculated metrics: homogeneity score, completeness score, v measure, adjusted Rand score, adjusted mutual score, Jaccard score, silhouette score (Bray-Curtis metric), inertia and entropy. Number of clusters k goes from 2 to maximum 10. See Figure 1 for a graphical visualization of silhouette score, Jaccard score and entropy

Supplementary Material 2. Supplementary Table 2. HIVE gene selection. Information on genes in common between the two selection methods and GeneIDs of genes selected by HIVE and meta-analysis

Supplementary Material 3. Supplementary Table 3. GO-term enrichment analysis for HIVE and meta-analysis. For each PlantGoSlim GO-term (Term) found enriched from hypergeometric test (*p*-value<= 0.05), in both HIVE and meta-analysis (META) selection, it is reported the corresponding p-value (method-pval) and the list of genes associated to the respecrive GO-term (method-entries).

Supplementary Material 4. Supplementary Table 4. Gene association to condition for HIVE and meta-analysis. HIVE and meta-analysis (META) selected genes, each associated to a specific or multiple conditions in the integrated dataset. ad_bio and as_bio = *Meloidogyne arenaria* (*A. duranensis* & *A.stenosperma*), ad_abio and as_abio = Drought stress (*A. duranensis* & *A.stenosperma*), as_cross = *Meloidogyne arenari*a (*A. stenosperma*)+Drought stress.

Supplementary Material 5. Supplementary Table 5. Kolmogorov-Smirnov test p-values for each pair of log2 fold-change cumulative distributions.Stress condition for gene association (specific or shared to all stresses), and the pair of cumulative distributions taken into consideration for the test. bkg = background log2FC distribution, while HIVE and META (meta-analysis) = considered log2FC comes from the gene selection of the corresponding tool

Supplementary Material 6. Supplementary Table 6. Validated or qRT-PCR confirmed genes in our study.The FAIR representation of results from a literature screening of studies with similar stress conditions to our case-study. When gene accession names for *Arachis duranensins* where not directly available but only qRT-PCR for genes in *Arachis stenosperma* were reported, also the orthogroups from OrthoFinder application are included. Columns refers to: Reference study/s including this one when the tailored analysis were needed; gene symbol or abbreviation reported in reference studies; gene accession ID for Arabidopsis thaliana identified by Martins et al., 2022; *A. stenosperma* gene ID in PeanutBase; *A. stenosperma* gene ID in GeneBank; Corresponding orthogroup (suffix “_Arast” identify orthogroups found in this study while “_Athal” from previous study of Martins et al., 2022); *A. duranensis* gene ID or orthologues list; GeneID found in our list of genes selected with HIVE application.

Supplementary Material 7. Supplementary Table 7. Transcription factors' families in HIVE regulome. For each transcription factor (TF) family found in HIVE selection, it is shown the number and the name of genes belonging to the family. The TF families are retrieved by the public database PlantTFDBv5.0

Supplementary Material 8. Supplementary Table 8. Transcription factors’ families in GRN communities.Number and percentage of occurrence of each transcription factor (TF) family in each of the 19 GRN communities. Comm = community, n. = number of TF in the corresponding family

Supplementary Material 9. Supplementary Table 9. Phytohormones related genes in GRN communities. Number and percentage of occurrence of each phytohormone related pathway in each of the 19 GRN communities. Comm = community, n. = number of phytohormones related genes in the corresponding pathway

Supplementary Material 10. Supplementary Table 10. Number of NBS-LRR genes in GRN communities.The number and the gene name of each NBS-LRR gene in each of the 19 GRN communities

Supplementary Material 11

## Data Availability

All data are available in this manuscript and HIVE scripts can be found in the GitHub repository [https://github.com/Plant-Net/HIVE.git] (https:/github.com/Plant-Net/HIVE.git).
