# Supplementary material for: Disentangling Arachis response to biotic and abiotic stress using multi-transcriptomics integration
Source: BMC Plant Biol. 2026 Mar 13;26:728. doi: 10.1186/s12870-026-08551-5 (PMC13101102; doi:10.1186/s12870-026-08551-5)
Supplement: Supplementary file 11 — Supplementary Material 11 [file 12870_2026_8551_MOESM11_ESM.docx]

**
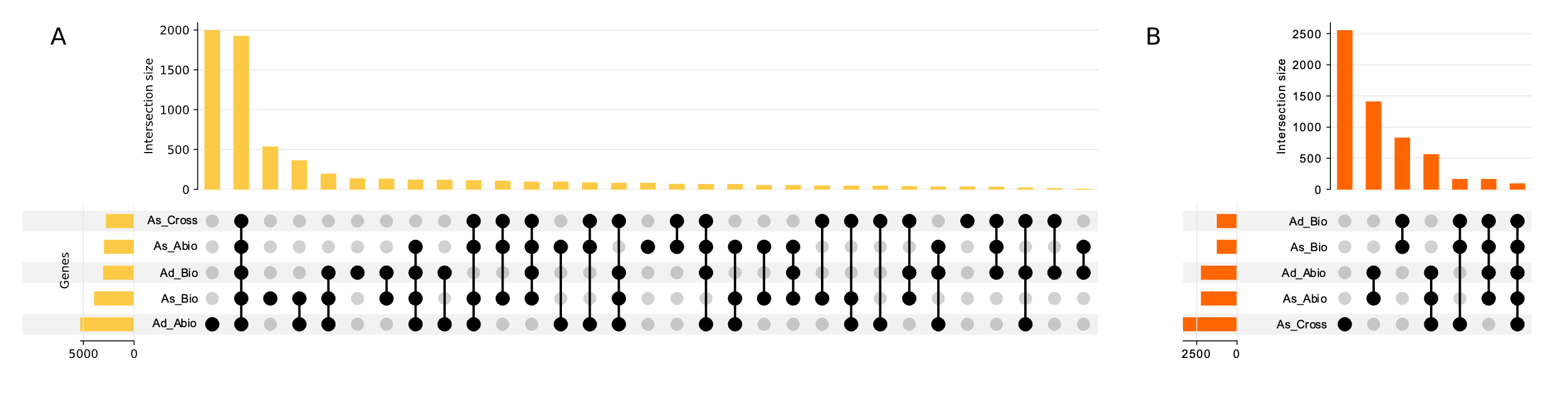
**

**Supplementary Figure 1. Combination of shared and specific signature selected by HIVE or meta-analaysis.**

A) Upset plot of HIVE selected genes representing all possible combinations of gene-association to condition. Conditions are distinguished between peanut species and type of stress. B) Same visualization but for meta-analysis selected genes.

*
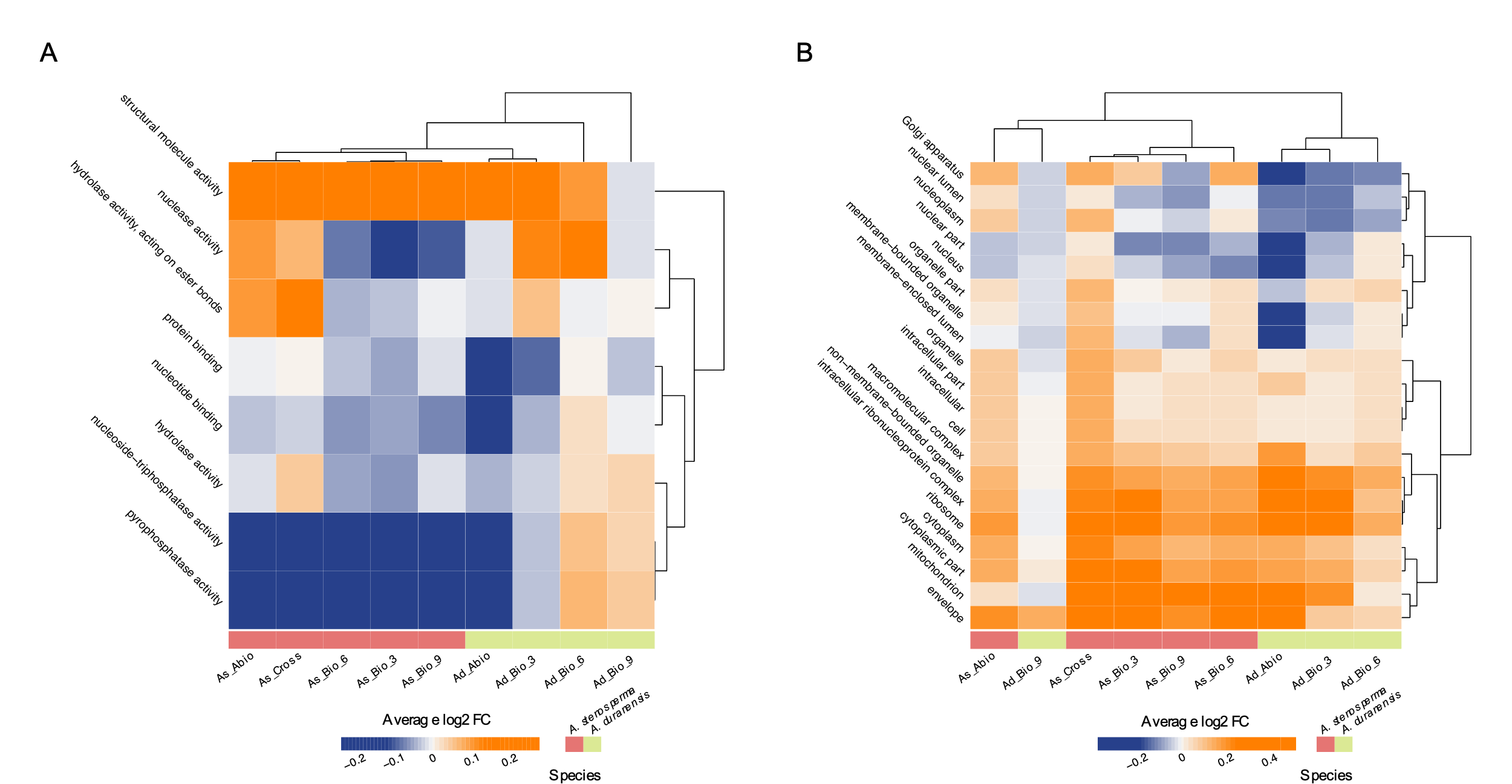
*

**Supplementary Figure 2. Enriched GO-terms from functional analysis of HIVE selected genes associated to common conditions.**

Enriched GO-terms (p-value <= 0.05) in selected genes associated to common conditions for A) Molecular Function ontology category B) Cellular Component. The “_3”, “_6” and “_9” notation refers to 3, 6, and 9 days post infection.

**Supplementary Figure 3. Transcription factors families and phytohormones associated to specific and all stress condition.**

Expression pattern profile and proportion of selected genes related transcription factor (TF) families and phytohormones found to be modulated in only biotic conditions, only abiotic and/or in all conditions. A) Heatmap of log2 fold-change expression patterns of TFs found to be modulated in all conditions. B) Heatmap of log2 fold-change expression for phytohormones in the same set of gens. A hierarchical clustering is made on gene expression profiles for both heatmaps to better distinguish modulation patterns of considered genes. C) Proportions of TFs important stress related families in only biotic, only abiotic and all conditions. D) Same representation but for phytohormones.

**Supplementary Figure 4. Expression profiles for transcription factors and their targets in Gene Regulatory Network by integrated analysis.**

A) Heatmap of log2 fold-change expression profiles of bZIP TF (Aradu.C0RFP) and its targets, involving also uncorrelated expression with the Ethylene-related gene Aradu.MA5U7. B) Correlated log2 fold-change expression profiles of ERF TF (Aradu.K41I0) and its targets, including Jasmonic Acid related gene Aradu.1C9UI.

**
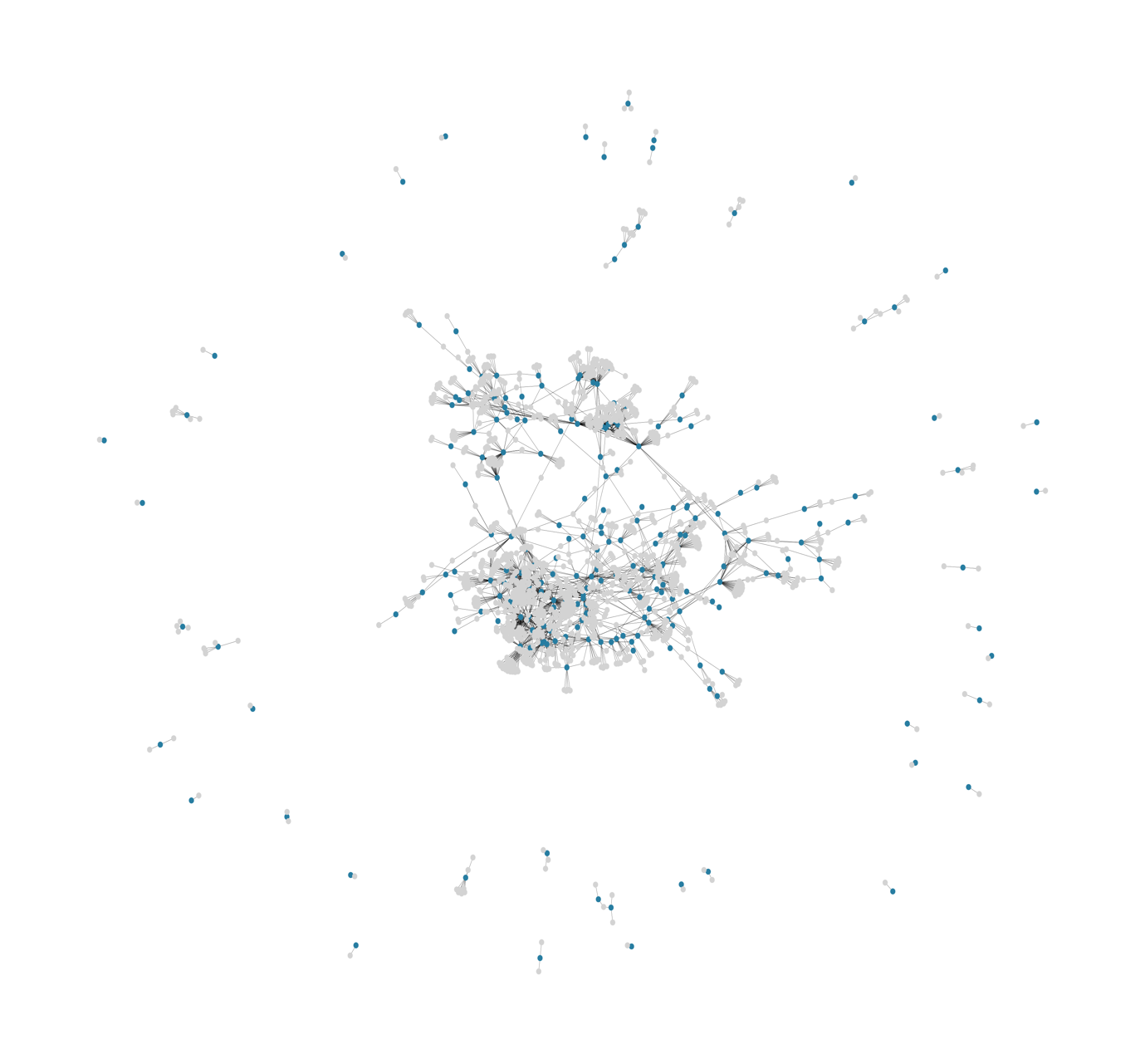
**

**Supplementary Figure 5. Global inferred regulome.**

The comprehensive Gene Regulatory Network inferred by considering the remaining 224 transcription factors as “regulators”. The blue nodes represent the regulators and the grey nodes their target genes.


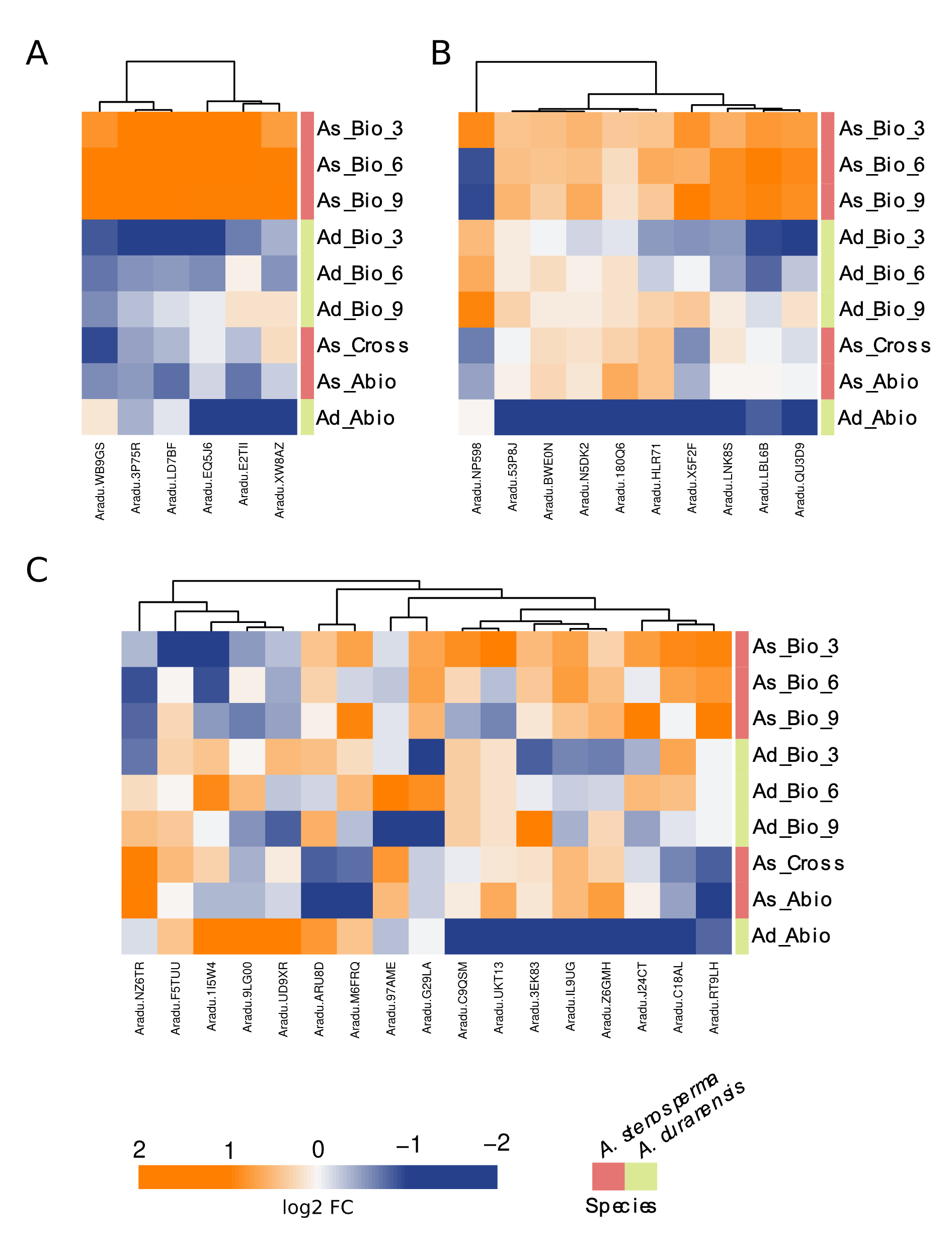


**Supplementary Figure 6. Expression profiles of genes in Gene Regulatory Network communities.**

A) Log2 fold-change expression profiles of ERF TFs and hormones related genes in community 7. B) Log2 fold-change expression profiles of bHLH TF and NBS-LRRs in community 8. C) Log2 fold-change expression profiles of FAR1 TF and NBS-LRR found in community 14.

**Supplementary Table 1. K-means clustering performances.**

The clustering performances for the four dataset setups (Raw Data, Latent Space, PCA, t-SNE). Calculated metrics: homogeneity score, completeness score, v measure, adjusted Rand score, adjusted mutual score, Jaccard score, silhouette score (Bray-Curtis metric), inertia and entropy. Number of clusters k goes from 2 to maximum 10. See Figure 1 for a graphical visualization of silhouette score, Jaccard score and entropy.

**Supplementary Table 2. HIVE gene selection.**

Information on genes in common between the two selection methods and GeneIDs of genes selected by HIVE and meta-analysis.

**Supplementary Table 3. GO-term enrichment analysis for HIVE and meta-analysis.**

For each PlantGoSlim GO-term (Term) found enriched from hypergeometric test (p-value <= 0.05), in both HIVE and meta-analysis (META) selection, it is reported the corresponding p-value (method-pval) and the list of genes associated to the respecrive GO-term (method-entries).

**Supplementary Table 4. Gene association to condition for HIVE and meta-analysis.**

HIVE and meta-analysis (META) selected genes, each associated to a specific or multiple conditions in the integrated dataset. ad_bio and as_bio = *Meloidogyne arenaria* (*A. duranensis* & *A.stenosperma*), ad_abio and as_abio = Drought stress (*A. duranensis* & *A.stenosperma*), as_cross = *Meloidogyne arenari*a (*A. stenosperma*)+Drought stress.

**Supplementary Table 5. Kolmogorov-Smirnov test p-values for each pair of log2 fold-change cumulative distributions.**

Stress condition for gene association (specific or shared to all stresses), and the pair of cumulative distributions taken into consideration for the test. bkg = background log2FC distribution, while HIVE and META (meta-analysis) = considered log2FC comes from the gene selection of the corresponding tool.

**Supplementary Table 6. Validated or qRT-PCR confirmed genes in our study.**

The FAIR representation of results from a literature screening of studies with similar stress conditions to our case-study. When gene accession names for *Arachis duranensins* where not directly available but only qRT-PCR for genes in *Arachis stenosperma* were reported, also the orthogroups from OrthoFinder application are included. Columns refers to: Reference study/s including this one when the tailored analysis were needed; gene symbol or abbreviation reported in reference studies; gene accession ID for Arabidopsis thaliana identified by Martins et al., 2022; *A. stenosperma* gene ID in PeanutBase; *A. stenosperma* gene ID in GeneBank; Corresponding orthogroup (suffix “_Arast” identify orthogroups found in this study while “_Athal” from previous study of Martins et al., 2022); *A. duranensis* gene ID or orthologues list; GeneID found in our list of genes selected with HIVE application.

**Supplementary Table 7. Transcription factors' families in HIVE regulome.**

For each transcription factor (TF) family found in HIVE selection, it is shown the number and the name of genes belonging to the family. The TF families are retrieved by the public database PlantTFDBv5.0.

**Supplementary Table 8. Transcription factors’ families in GRN communities.**

Number and percentage of occurrence of each transcription factor (TF) family in each of the 19 GRN communities. Comm = community, n. = number of TF in the corresponding family.

**Supplementary Table 9. Phytohormones related genes in GRN communities.**

Number and percentage of occurrence of each phytohormone related pathway in each of the 19 GRN communities. Comm = community, n. = number of phytohormones related genes in the corresponding pathway.

**Supplementary Table 10. Number of NBS-LRR genes in GRN communities.**

The number and the gene name of each NBS-LRR gene in each of the 19 GRN communities.
